# *PyramidalExplorer*: A New Interactive Tool to Explore Morpho-Functional Relations of Human Pyramidal Neurons

**DOI:** 10.3389/fnana.2015.00159

**Published:** 2016-01-06

**Authors:** Pablo Toharia, Oscar D. Robles, Isabel Fernaud-Espinosa, Julia Makarova, Sergio E. Galindo, Angel Rodriguez, Luis Pastor, Oscar Herreras, Javier DeFelipe, Ruth Benavides-Piccione

**Affiliations:** ^1^Universidad Rey Juan CarlosMadrid, Spain; ^2^Center for Computational Simulation, Universidad Politécnica de MadridMadrid, Spain; ^3^Laboratorio Cajal de Circuitos Corticales, Centro de Tecnología Biomédica, Universidad Politécnica de MadridMadrid, Spain; ^4^Instituto Cajal, Consejo Superior de Investigaciones CientíficasMadrid, Spain; ^5^Departamento de Arquitectura y Tecnología de Sistemas Informáticos, Universidad Politécnica de MadridMadrid, Spain

**Keywords:** visual analysis, dendritic spines, cerebral cortex, functional models, 3D reconstructions

## Abstract

This work presents *PyramidalExplorer*, a new tool to interactively explore and reveal the detailed organization of the microanatomy of pyramidal neurons with functionally related models. It consists of a set of functionalities that allow possible regional differences in the pyramidal cell architecture to be interactively discovered by combining quantitative morphological information about the structure of the cell with implemented functional models. The key contribution of this tool is the morpho-functional oriented design that allows the user to navigate within the 3D dataset, filter and perform Content-Based Retrieval operations. As a case study, we present a human pyramidal neuron with over 9000 dendritic spines in its apical and basal dendritic trees. Using *PyramidalExplorer*, we were able to find unexpected differential morphological attributes of dendritic spines in particular compartments of the neuron, revealing new aspects of the morpho-functional organization of the pyramidal neuron.

## Introduction

Over recent years, the field of neuroanatomy has evolved considerably, thanks to the use of classical techniques and the introduction of new procedures and powerful techniques to examine the organization of the nervous system (reviewed in DeFelipe, 2002, 2010). These new techniques have shifted the bottleneck of their workflow from acquisition to data analysis, generating a high volume of complex data to be examined. For example, the current development of semi- or automated 3D reconstruction and visualization methods to analyze synapses, individual neurons or large brain regions (or the whole brain in the case of the mouse) is of great importance as the large volume of data generated is critical to define brain connectivity and function, and to face the challenge of unraveling the extraordinary complexity of the nervous system in general.

Visualization-based approaches have proved to be successful in the analysis of complex systems, exploiting the ability of the human visual system to extract information from visual scenarios (Fuchs and Hauser, 2009; Keim et al., 2010; Munzner, 2014). Recently, Beyer et al. (2013) have developed *Connectome Explorer* that implements *ad hoc* algebra queries based on a set of operators and predicates of spatial, topological and attribute information. The information is extracted from a dataset of segmented structures of electron microscopy analyses. Other tools have been developed for the study of other levels of detail (i.e., relationships among neurons and brain parts) such as *Brain Gazer* (Bruckner et al., 2009) or *Neuron Navigator* (Lin et al., 2011).

Here we specifically developed a new tool to interactively explore and reveal the detailed organization of pyramidal neurons. Our tool —*PyramidalExplorer*— differs from others in that it does not analyze relationships among neurons in the way that, for example, *connectome explorer* would (i.e., by analyzing if a specific axon forms a synapse with the same dendrite or a different one, or whether synapses formed in the same dendrite occur sequentially or not). In the case of *PyramidalExplorer*, we explore possible *regional* differences in the pyramidal cell architecture by combining quantitative morphological information about the structure of the cell with implemented functional models. At present, the software displays an example of a human pyramidal neuron that was 3D reconstructed from high-resolution confocal stacks of images. However, the present application allows the input of different cell types, species and kinds of data (including electron microscopic reconstructions), thereby broadening the usage of the tool (see methods and results for further details).

Despite the fact that 3D light microscopic techniques are limited to a lower level of resolution, they remain the method of choice to obtain large-scale spatial information regarding the location of particular structures in the neuron like, for example, the distribution of putative synaptic connections along the dendrites of the cell. We focused on the microanatomy of pyramidal neurons, since they are the most abundant and characteristic neuronal type in the neocortex. Pyramidal neurons are the main projection neurons, since most of the processed information leaves the cortex through the axons of pyramidal cells to reach other cortical areas or subcortical nuclei. Also, their dendritic spines (for simplicity, spines) are the main postsynaptic target of excitatory glutamatergic synapses. In turn, pyramidal cell axons constitute the main source of these synapses (reviewed in DeFelipe and Fariñas, 1992). Thus, our understanding of the synaptic organization of the neocortex largely depends on the knowledge available regarding synaptic inputs to pyramidal cells.

At present, there are several software tools that allow the 3D reconstruction and visualization of microanatomical details of the neuron from confocal stacks of images. For example, Imaris software (Bitplane AG, Zurich, Switzerland) allows the generation and visualization of 3D surfaces of neuronal elements and displays several measurements from the reconstruction. However, *PyramidalExplorer* imports the features computed from any image segmentation software tool capable of generating data using standard spreadsheet formats since, unlike Imaris, it has not been designed as an image segmentation tool, but rather as an interactive exploratory software tool. Furthermore, Imaris software does not include —or does not accurately calculate— certain important morphological variables related to cell function. To address this issue, the tool proposed here uses a new approach to analyze 3D reconstructed structures introducing specifically calculated morphological variables that capture functional properties of the cell (i.e., certain morphological variables are implemented from mesh reconstructions and functional models are calculated from morphological data). Additionally, it incorporates a query driven analysis, which allows users to perform Content-Based Information Retrieval (CBIR) operations. These operations have proven to be a very useful tool when dealing with a large amount of multimedia information, due to the maturity reached by the techniques involved (Smeulders et al., 2000; Lew et al., 2006; Tangelder and Veltkamp, 2008; Hu et al., 2011; Juneja et al., 2015). Also, the present approach surpasses other developed tools regarding the implemented query interactions, as well as the interaction with both the raw data and the derived data extracted from the morphological information available in the meshes. Furthermore, no specific prior knowledge of the dataset is required to extract the derived data. These tasks have been integrated into a tool called *PyramidalExplorer*, which is publicly available at http://gmrv.es/pyramidalexplorer.

## Methods and Results

### Data Acquisition and Preparation

**FIGURE 1:**
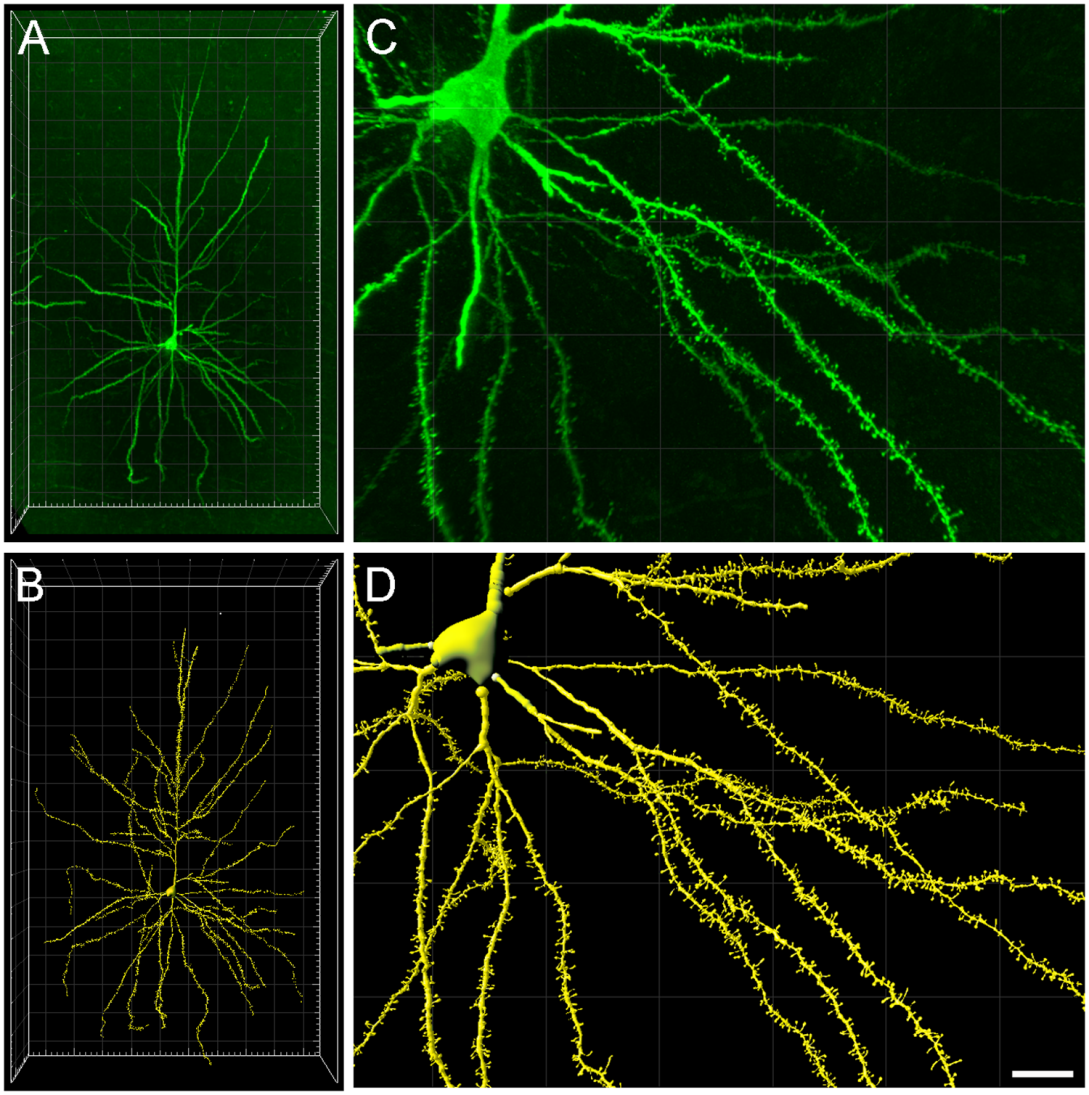
**(A)** Intracellularly injected layer III pyramidal neuron of the human cingulate cortex visualized in 3D from high-resolution confocal stacks of images. **(B)** Three-dimensional reconstruction of the complete morphology of the cell shown in **(A)**. **(C,D)** Higher magnification images of **(A,B)**, respectively, showing basal dendritic segments to illustrate the reconstruction of the dendritic shafts and spines. Scale bar (in **D**): 100 μm in **(A,B)** and 20 μm in **(C,D)**.

The input data consist of 3D reconstructed apical and basal arbors from a human pyramidal neuron that was intracellularly injected with Lucifer Yellow (LY) in layer III of the human cingulate cortex from a 40-year-old human male obtained at autopsy (2–3 h post-mortem). Further information regarding tissue preparation, injection methodology and immunohistochemistry processing is outlined in Benavides-Piccione et al. (2013). The injected cell was fully imaged at high magnification using tile scan mode in a Leica TCS 4D confocal scanning laser attached to a Leitz DMIRB fluorescence microscope (**Figure 1**). Fluorescent labeling profiles were imaged, using an excitation wavelength of 491 nm to visualize Alexa fluor 488. Consecutive stacks of images at high magnification (×63 glycerol) were acquired to capture dendrites along the apical and basal dendritic arbors. Since intracellular injection of the pyramidal cell was made in 300 μm-thick coronal sections, the part of the dendritic arbor nearest the surface of the slice from which the cell soma was injected (typically at a depth of∼30 μm from the surface) was lost. In addition, with this method, the apical dendrites that run for further than ∼900 μm from the soma were not filled with dye, and therefore apical tuft was not included in the analysis. Using a similar method of intracellular injection, Krimer et al. (1997) estimated that the reconstruction of neurons represented approximately two–thirds of the total dendritic arbor of pyramidal cells.

Dendritic shafts and spines were individually reconstructed in 3D from the high-resolution confocal stacks of images (**Figures 1C,D**) using Imaris 7.6.5, Filament Tracer module (Bitplane AG, Zurich, Switzerland). Over 9000 spines were reconstructed along 8693 μm of the pyramidal neuron dendritic length (4263 μm of basal dendrites, 540 μm of main apical, and 3890 μm of collateral dendrites). The morphological variables used from Imaris included Spine Volume, Spine Area, Spine Length and Spine Position in the x, y, z dimension. However, since Imaris software does not include —or does not accurately calculate— certain important morphological variables from the spines related to function such as minimum or maximum diameters, these had to be retrieved by post-processing the 3D meshes. These values included Spine Maximum Diameter and Spine Neck Mean Diameter. All these processes were programmed using *ad hoc* python scripts.

### Functional Modeling

The trans-membrane voltages and currents were calculated using GENESIS simulator (Bower and Beeman, 1998). For integration of the membrane dynamics, we used an exponential Euler (explicit) scheme with the fixed step, *dt* = 1 μs.

Based on the data provided by the 3D reconstruction of the spines described above, we modeled spines according to their values of Spine Maximum Diameter, Spine Neck Mean Diameter, Spine Length, and Spine Neck Length. Mushroom spines were considered as two lumped cylinders for the spine head and neck, respectively. The spine neck displayed diameters ranging from 0.175 to 1 μm and lengths between 0.38 and 4.37 μm; the spine head showed both a diameter and length between 0.43 and 1.04 μm. Stubby spines were constructed as a single cylinder with a diameter ranging from 0.44 to 1.15 μm and a length from 0.32 to 1.66 μm. Thin spines showing a small head or filopodia (long protrusions without clear head) were not included in the model. All spines were attached to a parent dendrite compartment of constant diameter and length: 3 and 20 μm, respectively. The membrane capacitance, C_m_, internal resistivity, R_i_, and membrane resistivity, R_m_, were set to 1 μF/cm^2^, 100 Ω.cm, and 20,000 Ω.cm^2^, respectively.

The synaptic currents were modeled by:

$$\begin{array}{c} I_{syn}(t)=g_{syn}\;(t)(V_{m}-E_{syn})\;\\ g_{syn}(t)={\hat{g}}_{syn}\;(t/\tau_{syn})\exp(1-t/\tau_{syn}),t>0 \end{array}$$

with τ_syn_ = 2 ms, and the reversal potential E_syn_ = 0 mV for excitatory glutamatergic input (Makarova et al., 2011). The synaptic conductances were homogenously distributed throughout the surface of all spine heads with a constant density of 132 S/m^2^.

The variation of the transmembrane potential V_m_ in spine heads was calculated as the peak depolarization from rest.

### Content-Based Information Retrieval Operation

A query driven analysis was incorporated into the tool to allow users to perform Content-Based Information Retrieval operations. CBIR systems focus on searching for data as a function of the real data content, automating the information extraction process from raw data (Smeulders et al., 2000), but the set of objects that the system is able to recognize is much broader in comparison with the classical recognition systems. CBIR systems transform the contents of the raw data to a signature. The signature is built from a set of features, also named primitives, computed from the contents of the raw data. This signature is the data used in the matching process with all the items stored in the dataset.

**FIGURE 2:**
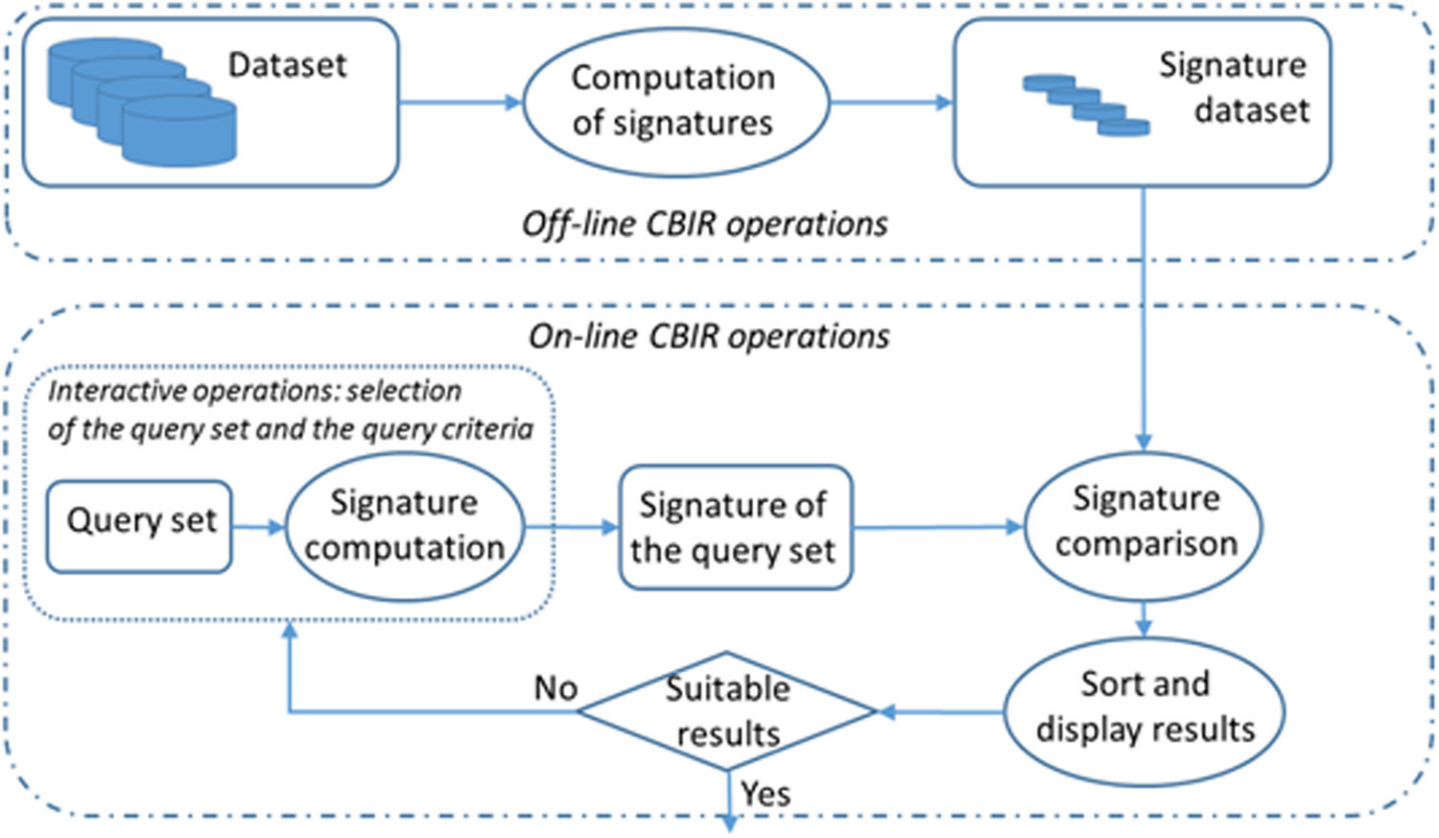
**Content-Based Information Retrieval (CBIR) system operation scheme**.

CBIR systems differ in terms of the features used to retrieve the information: text, color, texture, scheme, shape, volume, spatial restrictions, objective and subjective attributes, movement and cuts in videos, etc.; that is, any feature that can be used to represent information contents and has discriminant properties (Atrey and Hossain, 2010). Some other possible classification criteria for these kinds of systems include the way the image features are extracted –automatically, semi-automatically or manually–, the feature abstraction level or the independence between the different conceptual domains. **Figure 2** depicts the main operations involved in a CBIR system. This process has an off-line stage, which computes the signature of each item from the dataset. The system is then ready to process interactive queries following the steps below:

(1) Input/query introduction: The user first selects a set of items of the dataset to be used as a query reference and the system computes its signature. Then, query criteria are selected considering the most relevant features at any moment of the working session. These interactive operations allow the retrieval engine to compute the query signature.
(2) Query and dataset items’ signature comparison and sorting: The signature obtained in the previous stage is compared with all the dataset signatures and the system provides the user with a list of items sorted according to the criterion of greatest similarity to the query set. The volume of the computations depends on the size of the dataset.
(3) Results are displayed and the query image updates. The system presents a visual result of the sorted list to the user.

### The Graphical User Interface

**FIGURE 3:**
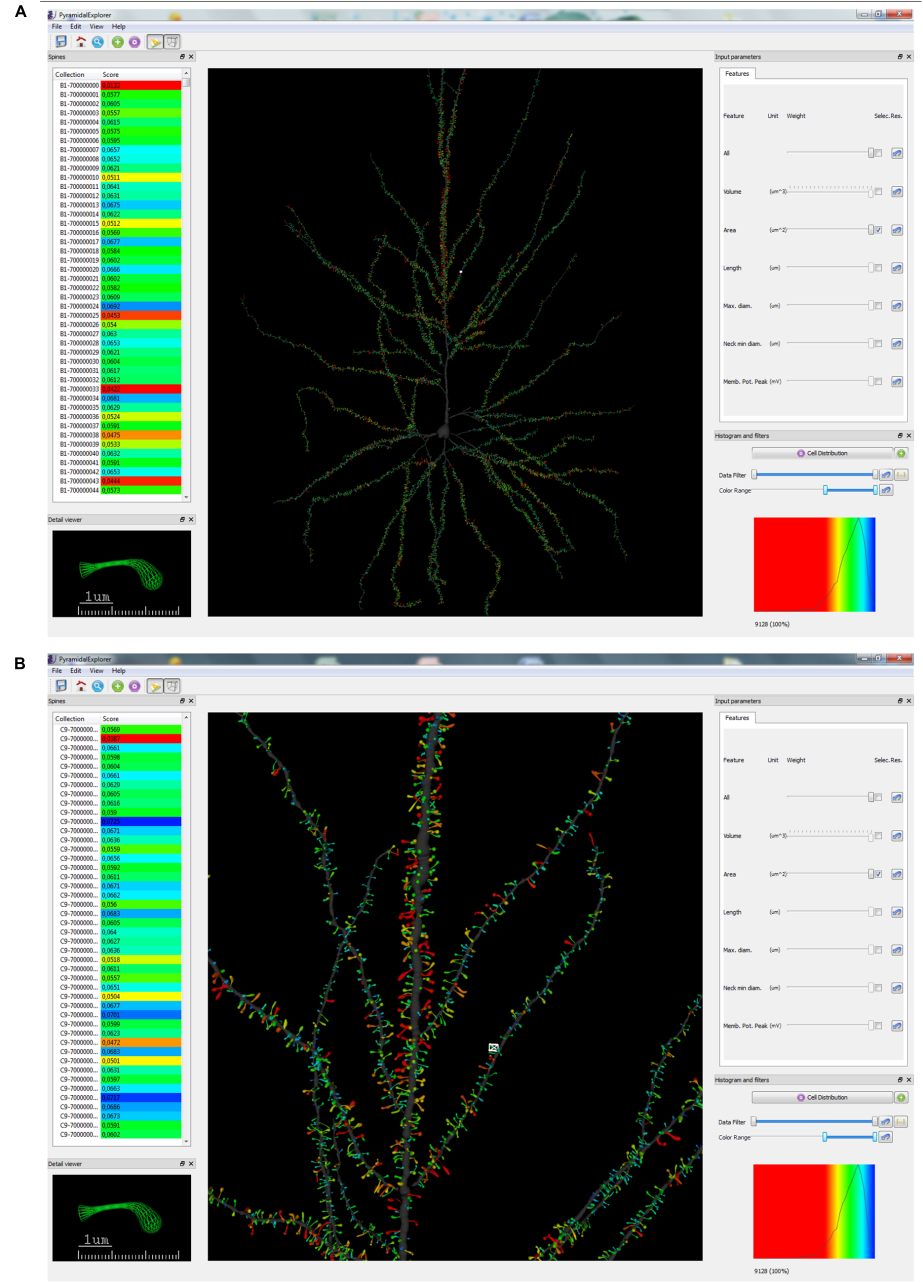
**Graphical user interface (GUI) of *PyramidalExplorer* showing the cell comparison query result concerning Spine Area, from the human pyramidal neuron in a global view **(A)** and in a zoom in view (B)**. Values are represented by color code. Red colors represent highest values whereas blue colors represent the lowest values. White frame in the main window highlights the spine visualized in the Detail viewer widget.

The graphical user interface (GUI) comprises a main window and four additional auxiliary widgets (**Figure 3A**). In the main window, *PyramidalExplorer* presents the 3D global view of the cell being studied. This view shows the original data, if no queries are performed yet, or the visual representation of the last scores obtained after launching a query. In this way, the users visualize all of the morphological elements of the neuron in the main window, obtaining a global view of the whole cell. Each individual structure is represented with a single 3D mesh stored in VRML files exported from *Imaris*. The user can interact with the whole scenario using the mouse for pan, rotate and zoom operations (**Figure 3B**).

The main window incorporates the following four auxiliary widgets:

• *Spines*. The purpose of this widget is to manage the list of spines sorting them according to different criteria.
• *Detail viewer*. Using this widget, the user can visualize in 3D the details of one spine selected from the collection under study.
• *Input parameters*. These allow the user to interact with the features included.
• *Histogram and filters*. The user can filter (Data filter) the results based on the histogram distribution. Also, adjustment for an optimal visualization of the results is available using Color range.

Any of the widgets can be resized and positioned at will, or disabled if, for example, there is not enough space on the screen. They can also be detached from the main window for separate management. The available menus and the tool bar are visible at the top of the screen. Users can select the neuron under study and save the results obtained after query or filter operations using the File menu. Also, the appearance of *PyramidalExplorer* can be customized using the View menu to select the background color, the color of dendritic shafts, the colormap used for visualizing the sorted results and the widgets that are active. Additional shortcuts are available for selecting the closed or the wireframe mesh representation, as well as for performing fast navigation actions like positioning over the selected spine or going back to the global view of the neuron. The tool bar allows users to perform the most frequent interactions.

**FIGURE 4:**
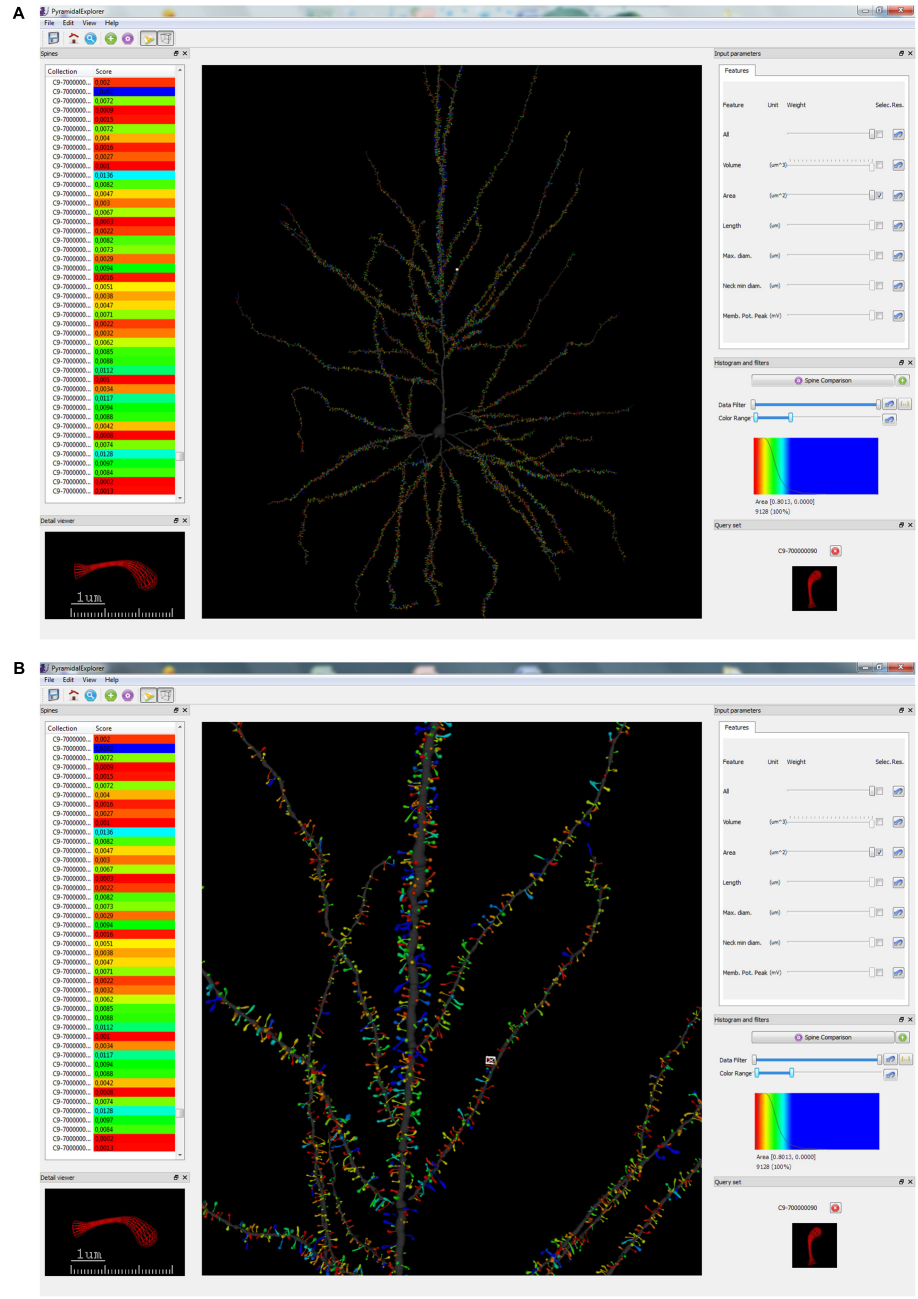
**Graphical user interface (GUI) of *PyramidalExplorer* showing the spine comparison query results concerning Spine Area, from the human pyramidal neuron in a global view **(A)** and in a zoom in view **(B)**.** Values are represented by color code. Red represents the most similar spines with respect to the query set (shown at the bottom, right-hand corner), while blue shows the most different spines. This query set specifically displays the comparison of one selected spine (which is also shown in the Detail viewer widget) with the most similar spines of the dataset. White frame in the main window highlights the spine visualized in the Detail viewer widget.

Each spine of the dataset has a unique name and the collection of names is listed in the Spines’ manager widget. When a query is performed, the Score column is displayed showing the similarity results obtained (see below for further explanation). The list can be reordered according to this value by simply clicking on the Score label. Color codes are also mapped in the Score column as a visual reference to highlight the level of similarity achieved with a particular score (see **Figures 3** and **4**). The original sorting based on alphabetical order can be obtained by clicking on the Collection label.

Regarding the Detail viewer widget, it can present either the original mesh exported by Imaris or the wireframe representation of this mesh. The same interaction controls with the mouse that are available for the 3D Global View are also implemented in this widget. *PyramidalExplorer* also provides a visual reference of the real size of the spines shown in this widget. This option is also accessed through the View menu as well as with the shortcut *Ctrl + m* (measure view). It is easy to return to the previous visualization by undoing this operation through the View menu or pressing the *Ctrl +m* shortcut again. Resetting to the default position is also available in this view by pressing the space bar. Hot keys can be used to speedup interaction if the user wishes to concentrate on the spine selected in the Detail viewer widget (*f* key) or if the 3D global view is required again (*a* key). Resetting to the default position is also achieved using the space bar.

In order to perform a query, users have controls to enable/disable and to assign weights to each of the morpho-functional features extracted. The weights can be set in the [-1,1] range using sliders and the importance of the selected features can be established, where positive values mean the user wants to find objects that are similar to the feature while negative values mean the user wants to find objects that are dissimilar to the feature. These controls are displayed in the Input parameters widget. For combining the different range of values available, a normalization stage is performed before computing the query score. After feature normalization, all of the scores will fit in the interval [0,1], allowing the objective function to be applied to the selected criteria as a set of summation terms.

Once the query parameters are selected, that is, the query set and the query criteria, users launch the query with the control button available in the Histogram and filters widget. There are two types of queries: the study of the distribution of a particular feature in the dendritic arbor (Cell Distribution) and the study of a particular element of the neuron (e.g., spine) with regard to the remaining elements (Spine Comparison). **Figure 3A** shows the result of applying a Cell Distribution query, concerning the Spine Area. In this case, one feature is selected and when the query is launched, the system returns all the values of the dataset for this feature, assigning color codes according to the relative position of the structure in the ranking of the feature values.

By contrast, **Figure 4A** shows the result of applying a Spine Comparison query to the spine area. This query specifically displays a comparison of the selected spine/s with the most similar spines of the dataset. In both cases, a histogram is displayed with the distribution of similarity values along the color scale selected. The range of score values is ordered according to the colormap selected (in the View menu). When queries are launched, the view updates its contents showing the similarity scores obtained with respect to the query set. **Figures 3A and 4A** show the whole dendritic tree, but shafts can be hidden if users want to focus only on the spines (i.e., assigning a transparent color to the dendritic shaft). Other customization options include changing the background color and choosing another colormap for the query results.

Users also have some filter control over the color scale. They have a double slider to adjust the active range of items in the dataset according to the ordered scores. Also, they have a control button to activate the complementary set of values of the current range defined with the slider. Another widget allows users to reduce the dynamic range of values under study, reassigning the maximum and minimum values visualized to the extreme colors of the visual mapping selected. This transformation enhances the visibility of certain subsets of interest for the analysis under consideration in the event that these items have very close values that are not visually noticeable with the whole distribution. Also, users can maximize the Histogram widget for a better examination of the query results.

When Spine Comparison is selected, the image of the query set defined by the user is displayed (**Figures 4A,B**; bottom, right-hand corner). In this case, there is a single spine in the query set, and therefore only one image is shown. However, it is also possible to define a Spine Comparison query set that includes several spines. If there are no items in this set, no images are presented. The color assigned to the spine in the Detail viewer widget (bottom, left-hand corner) is updated anytime a query is carried out.

### Viewing Differential Morphological and Functional Features of the Neuron

**FIGURE 5:**
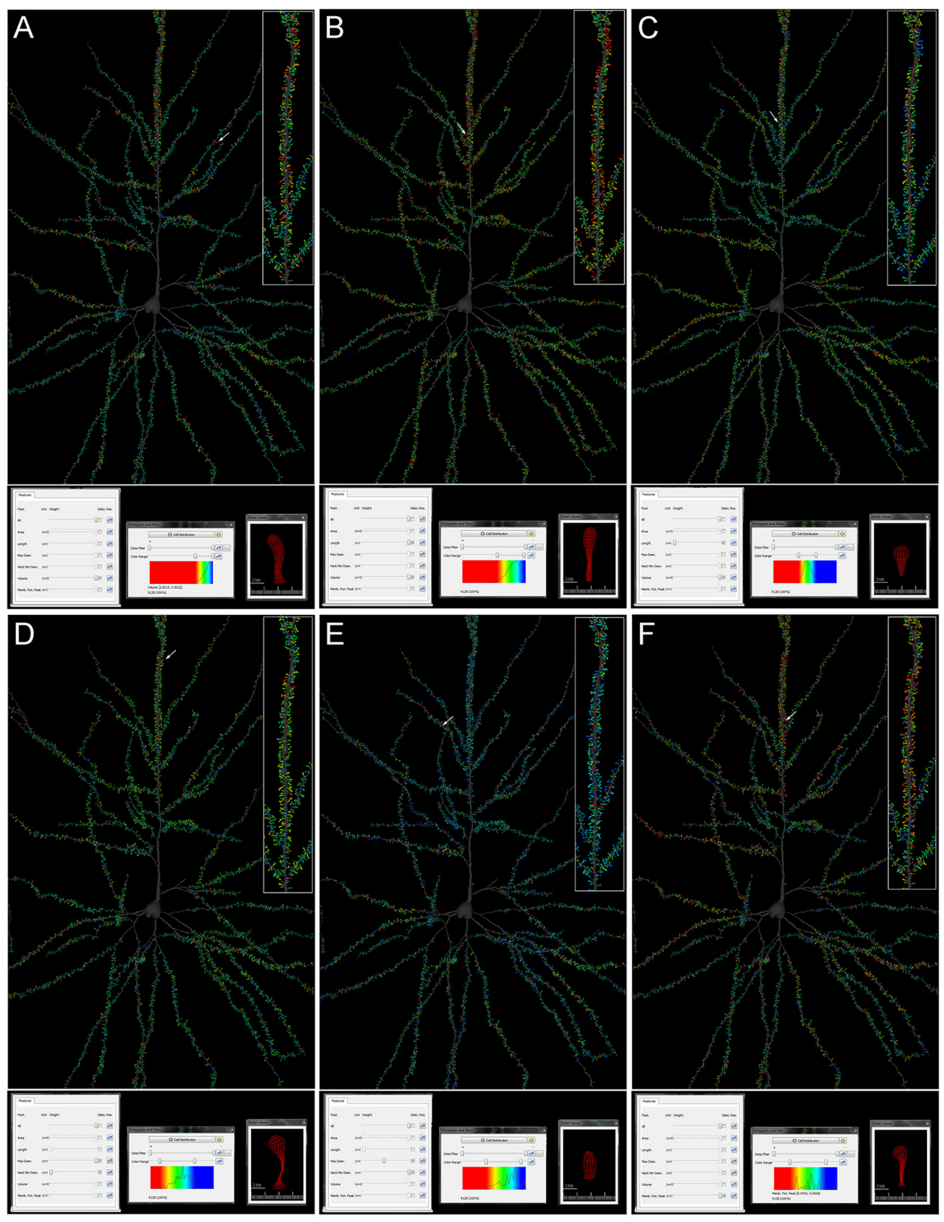
**Cell comparison query result from the human pyramidal neuron concerning Spine Volume **(A)**; Spine Volume and Length **(B,C)**; Maximum Diameter and Mean Neck Diameter **(D,E)**; and Membrane Potential Peak **(F)**.** Values are represented by color code. Red colors represent the highest values whereas blue colors represent the lowest values. Higher magnification images of the apical dendrites are shown on the right (inset). Arrows indicate representative spines of the highest values, which are also shown on the Detail viewer widget at the bottom right. See results for further details.

Here we present an example of differential morphological features of spines in particular compartments of the neuron. The morphological features extracted (Spine Volume, Spine Length, Spine Area, Spine Max Diameter, and Spine Neck Mean Diameter) allow possible regional differences in the pyramidal cell architecture to be visualized easily. For example, we can interactively discover that the spines with the largest volumes are found in the main apical dendrite, while some —but not all— collateral and basal dendrites have a particularly high percentage of large spines (**Figure 5A**). If we additionally select the Length feature, we can visualize the position of the largest and longest spines (**Figure 5B**), which is different from the position of the largest and shortest spines (**Figure 5C**). As another example, if we select Maximum Spine Diameter and Spine Neck Mean Diameter, we can visualize the position of the spines with the largest heads and the thinnest necks (**Figure 5D**), which have a different distribution in the arbor from those which do not have the largest heads and have the thickest necks (**Figure 5E**). Also, functional information was generated off-line and linked to each spine, by combining quantitative morphological information about the structure of the cell with implemented functional models. By doing this, it was possible to easily visualize the estimation of the membrane potential peak that each spine would generate in the cell. As shown in **Figure 5F**, particular dendrites display different membrane potential peak values, as do particular dendritic segments within the same dendrite.

This is an example of a human pyramidal neuron that was 3D reconstructed from high-resolution confocal stacks of images. However, the present application can manage different kinds of data, including electron microscopic reconstructions. Instructions for loading your own data into *PyramidalExplorer* are available at http://gmrv.es/pyramidalexplorer.

*PyramidalExplorer* is also intended to grow as a bidirectional tool to assist or link to others, including software specifically addressing functional exploration, such as biophysical modeling requiring anatomical details of neurons. The way to implement data transfer between tools shall be developed on demand. In this paper, we fed the anatomical details of real spines obtained by *PyramidalExplorer* into an off-side computational environment and the results were fedback to ease visualization. For example, you may choose to visualize the amplitude of voltage changes at the spine head in individual spines or by levels of amplitude by toggling the Membrane Pot. Peak feature. The biophysical parameters (conductance density) cannot be obtained with experimental techniques, but can be calculated using anatomo-functional relations already established in the literature (spine head size and AMPA receptor density). This illustrates the possibilities of functional exploration but, clearly, specific routines may enable, for instance, the selection of particular groups of spines for their activation in bound computational software (Neuron, Genesis, etc.).

## Discussion

The development of the tool *PyramidalExplorer* allowed us to interactively explore and reveal new aspects of the morpho-functional organization of the human pyramidal neuron. In particular, spines showed differential morphological attributes in different compartments of the neuron, reflecting regional variations in the functional properties of the cell.

Regarding the technologies used in the implementation, we followed a user-centered design methodology (UCD) because it is the strategy that best suits the problem of applying content-based retrieval methods to users who had no previous experience using this kind of technique. It should be noted that we did not find any appropriate exploratory tool for the problem at hand. The implementation was carried out following an agile development method similar to scrum (Rising and Janoff, 2000) since this approach fits in very well with UCD principles. It was based on small sprints, each one defined by a specific goal resulting in a new improvement of the prototype. Different basic milestones were defined for the different stages of the development, covering different aspects like GUI and usability improvements; visualization and perception issues; tuning of the retrieval engine; filtering; and 3D navigation capabilities. Another important objective was to assure the portability of the software tool, that is, to check that it could be used in the most common operating systems currently used. *PyramidalExplorer* was successfully tested in different versions of Windows, Mac OSX, and Linux, covering both x86 (32 bits) and x64 (64 bits) architectures.

*PyramidalExplorer*’s implementation was based on two well-tested and freely distributed software toolkits. Our software tool has been designed to operate within the same free distribution policy. The interface was built using Qt^1^ (vs. 4.8), which is a library that is used the world over, offering both stability and portability over a wide range of software and hardware platforms, as well as providing all the widgets and controls that users need and meeting all usability requirements. Additionally, Open Scene Graph^2^ was used for rendering duties. This toolkit facilitates some rendering and interaction processes and, taking into account that it uses OpenGL^3^, it also provides flexibility and stability in the implementations. It currently comes with an example of a human cell dataset which will allow potential users to test the tools and appreciate the advantages of the approach and techniques presented in this paper. However, users are able to access a web portal to convert their own datasets and load them into *PyramidalExplorer*.

The straightforward visualization of a large number of different functionally oriented variables of the cell allowed the extraction of meaningful information. For example, the visualization of variations in the number of spines in the different regions of the neuron is practically equivalent to the total number of excitatory synaptic inputs to that pyramidal neuron, since most glutamatergic excitatory synapses that pyramidal cells establish are on their spines and the vast majority of spines establish at least one glutamatergic excitatory synapse (see DeFelipe and Fariñas, 1992; Arellano et al., 2007a). Also, variations in the size and shape of the spine reflect distinct functional properties of the spine. Quantitative analyses have demonstrated strong correlations between the spine morphology and its synaptic structure. Specifically, the spine head volume in the neocortex is correlated with the area of the PSD (Arellano et al., 2007b). Moreover, PSD area is correlated with the number of presynaptic vesicles, the number of postsynaptic receptors and the ready-releasable pool of transmitter. By contrast, the length of the spine neck is proportional to the extent of biochemical and electrical isolation of the spine from its parent dendrite (Harris and Stevens, 1989; Yuste and Denk, 1995; Nusser et al., 1998). More recent experiments have shown that larger spines can generate larger synaptic currents than smaller spines (Matsuzaki et al., 2004). Further studies show that spines are dynamic structures and fluctuations in spine morphology seem to mediate evoked and experience-dependent synaptic plasticity (Van Harreveld and Fifkova, 1975; Holtmaat and Svoboda, 2009; Araya et al., 2014; Tønnesen et al., 2014) and have important implications for cognition and memory (Kasai et al., 2010). Using the present tool, it was possible to easily observe that spine volumes and areas from apical dendrites were different from those of collateral compartments of apical arbors and/or basal compartments. Additionally, some basal dendrites showed regional morphological differences. This is in line with previous studies that reported that different domains of the basolateral dendrites have different properties with respect to afferent connectivity, plasticity and integration rules (Markram et al., 1997; Häusser and Mel, 2003; Gordon et al., 2006; Gelfo et al., 2009; Petreanu et al., 2009).

A significant contribution of this tool is the morpho-functional oriented design, which combines the quantitative morphological information of the structure of the cell with functional models that were specifically implemented from mesh reconstructions. These calculations revealed membrane potential peak that each spine would generate, based on the maximum and minimum spine diameters (see Methods and Results for further details). While these estimations were made off-line and manually entered as functional information linked to specific spines, a future upgrade may include the automated transfer of morphological and electrophysiological information between *PyramidalExplorer* and program simulators and platforms for the theoretical investigation of single cell and population electrophysiology requiring realistic morphological data (e.g., Neuron, Hines and Carnevale, 1997; Genesis, Bower and Beeman, 1998; BlueBrain). Thus, researchers would have access to libraries of real (or imaginary) neurons with thousands of spines and, after loading back the functional information obtained for each individual spine, they could visualize the role of specific groups of spines and spine morphologies in subcellular electrogenesis. Indeed, a number of experimental and theoretical results point to local dendritic spikes initiated by the different grouping of coactive spines as a major mechanism for (a) output decision and spike regime (Larkum et al., 2001; Ibarz et al., 2006; Katz et al., 2009), (b) the formation of independent computational dendritic subunits (Behabadi and Mel, 2014), and (c) the establishment of synaptic plasticity (Bar-Ilan et al., 2011). The 3D information contained in uploaded neurons may also benefit researchers investigating the role of spine morphology and location in the pathway-specific contribution to local field potentials and the EEG (Herreras et al., 2015). This should notably improve present models that normally use a spiny model cells (Lindén et al., 2011) and should lead to the discovery of important rules by explicit modeling of spines, which are the main contributors of electric current to the extracellular space.

This tool, therefore, opens a new opportunity to study and analyze the morphology of neurons and explore possible clustering or particular morpho-functional distributions of spines within the cell through Content-Based Retrieval operations. Using these operations, it is possible to find the spines that are alike and dissimilar within the neuron, according to particular morphological or functional variables. Furthermore, it is also possible to set a query using multiple morphological and/or functional variables and obtain data about which spines of the neuron present the most similar or dissimilar characteristics to a particular spine/s selected. Finally, there is considerable interest in examining the possible alterations to pyramidal cells associated with brain diseases, and their role in memory, learning and cognition (Fiala et al., 2002; Benavides-Piccione et al., 2004; Spruston, 2008; Luebke et al., 2010; Penzes et al., 2011). Thus, the application of *PyramidalExplorer* to study pyramidal cells in brain diseases —or their alterations after experimental manipulations in animal models— is expected to provide a new set of microanatomical data that will facilitate a better understanding of the alterations of pyramidal cells under these circumstances.

## Author Contributions

RB-P and IF-E performed tissue preparation, injection methodology, immunohistochemistry processing and 3D morphological reconstructions. JM and OH performed functional modeling. PT, OR-S, SG, AR performed content-based information retrieval operations and graphical user interface. PT, OR-S, IF-E, JM, AR, LP, OH, JD, and RB-P wrote the paper.

## Conflict of Interest Statement

The authors declare that the research was conducted in the absence of any commercial or financial relationships that could be construed as a potential conflict of interest.

## Footnotes

1 http://www.qt.io

2 http://www.openscenegraph.org

3 http://www.opengl.org
